# Siliceous spicules enhance fracture-resistance and stiffness of pre-colonial Amazonian ceramics

**DOI:** 10.1038/srep13303

**Published:** 2015-08-27

**Authors:** Filipe Natalio, Tomas P. Corrales, Stephanie Wanka, Paul Zaslansky, Michael Kappl, Helena Pinto Lima, Hans-Jürgen Butt, Wolfgang Tremel

**Affiliations:** 1 Institut für Anorganische Chemie und Analytische Chemie, Johannes Gutenberg-Universität Duesbergweg 10-14, 55099 Mainz, Germany; 2 Institut für Chemie, Martin Luther Universität Halle-Wittenberg, Kurt-Mothes Str. 2, 06120 Halle (Saale) Germany; 3 Max Planck Institut für Polymerforschung, Ackermannweg 10, 55128 Mainz, Germany; 4 Instituto de Alta Investigación, Universidad de Tarapacá, Casilla 7-D Arica, Chile; 5 Julius Wolff Institut, Charité- Universitätsmedizin Berlin, Augustenburger Platz 1, 13353 Berlin, Germany; 6 Museu Paraense Emílio Goeldi (MPEG), Av. Perimetral 1901, Terra Firme, 66070-530, Belém, Brasil

## Abstract

Pottery was a traditional art and technology form in pre-colonial Amazonian civilizations, widely used for cultural expression objects, utensils and as cooking vessels. Abundance and workability of clay made it an excellent choice. However, inferior mechanical properties constrained their functionality and durability. The inclusion of reinforcement particles is a possible route to improve its resistance to mechanical and thermal damage. The Amazonian civilizations incorporated freshwater tree sponge spicules (*cauixí*) into the clay presumably to prevent shrinkage and crack propagation during drying, firing and cooking. Here we show that isolated siliceous spicules are almost defect-free glass fibres with exceptional mechanical stability. After firing, the spicule Young’s modulus increases (from 28 ± 5 GPa to 46 ± 8 GPa) inferring a toughness increment. Laboratory-fabricated ceramic models containing different inclusions (sand, glass-fibres, sponge spicules) show that mutually-oriented siliceous spicule inclusions prevent shrinkage and crack propagation leading to high stiffness clays (*E* = 836 ± 3 MPa). Pre-colonial amazonian potters were the first civilization known to employ biological materials to generate composite materials with enhanced fracture resistance and high stiffness in the history of mankind.

The concept of combining materials with different properties such as composites goes back to prehistoric times. Given its natural abundance and workability, clay has been the material of choice for producing utilitarian pottery and cultural expression ornaments, as extensively documented by archaeological records^1^. However, the inferior mechanical properties of clay created the need to incorporate inclusions either inorganic (e.g. sand, sponge siliceous spicules, ground potsherds, mica, grog, ashes) or organic materials (e.g. grass, dung or straw) to reduce shrinkage and enhance fracture resistance^1^,^2^. For archaeologists and anthropologists, the nature of the inclusion material is a blueprint of the habits and traditions of a particular civilization^3^,^4^. Whereas the use of organic inclusions generates relatively large voids after firing which arrests crack propagation under thermal stress - e.g. cooking vessels^5^ - the functionality of inorganic inclusions remains largely unexplored, in particular, those of biological origin (mainly siliceous spicules and tree ashes).

The extensive pre-colonial Amazonian pottery archaeological record shows a clear and intended use of inorganic inclusions such as siliceous spicules of tree sponge (Demospongiae, *Drulia sp.*) (*cauixí*), grounded ashes of bark from *Licania sp. (caraipé)*, as well as shells and grog^3^,^6^,^7^,^8^,^9^,^10^,^11^,^12^,^13^. The use of *cauixí* was very spread throughout Amazonia in pre-Columbian ceramist occupations (ca. 1,500 B.C. up to the conquest time), although its use seems to have decreased or even abandoned in many areas over time^13^,^14^. The reason for this technological and cultural “*switch*” is still unclear. Today only a very few indigenous or traditional potters use this technology.

*Demospongiae* have skeletons made of micron-sized spicules, consisting of amorphous hydrated silica deposited around a proteinaceous axial filament^15^,^16^. The spicule function may vary from a predator deterrent to providing bulk mechanical rigidity^16^,^17^,^18^. Although spiculogenesis in *Demospongiae* is well established^16^, their mechanical properties are not well known due to their size and the lack of hierarchical structure, which is different from glass sponges (*Hexatinellida*). The complex hierarchical structure of the glass sponges entails mechanical properties that are extraordinary for silica-based materials. High flexibility, strength and toughness can be attributed to the presence of dissimilar structures at different length scales^19^. The values of the elastic (Young’s) modulus and the hardness of the individual sponge spicules are comparable to those of fused silica (e.g. in glass fibres). At the same time, they are highly flexible because their elastic modulus and hardness shows a centrifugal gradient across the spicule’s long axis^20^. Moreover, hydration appears to affect the mechanical properties of the spicules - in their natural environment. Dry spicules have a higher Young’s modulus and an equally higher stress to failure than hydrated specimens^21^.

Here we show that pre-colonial Amazonian potsherds contain highly oriented micrometer size siliceous spicules attributed to the species *Drulia uruguayensis (Demospongiae*, Porifera) - locally known as *cauíxi*. Taxonomically related and morphologically/structurally identical siliceous spicules from *Suberites domuncula* (*Demospongiae*, Porifera) were used as a model to recreate pre-colonial potsherds (see Supplementary Information Fig. S1). These siliceous spicules exhibit superior mechanical properties after firing (500 °C for 1 h) and behave like almost defect-free glass fibres. Using coil-roll Amazonian techniques (from Amazonian locals that still preserve this tradition), we have fabricated ceramic composite models to address the effect of inclusions (sand, glass fibres and siliceous spicules) on the final properties. We found that the shear force produced by the coil-roll technique induces siliceous spicule orientation, providing high stiffness and preventing crack propagation in comparison with other inclusions. The combination between preferentially orientated almost defect-free biological glass fibres and with a poor mechanical ceramic matrix to generate mechanically enhanced composite materials has been considered the blueprint of a creative process and successful technological implementation lead by pre-colonial South American civilizations.

Figure 1a shows a representative cracked potsherd sample (orange, 7 × 3 cm, vase wall, N1049/E880, Santa Helena archaeological site), where glassy needle-like structures with diameters of ≈10 μm and lengths of 100–200 μm were observed under a low magnification microscope (Fig. 1b). This feature, as seen after SEM inspection, is found in all Amazonian potsherds (Fig. 1c and see Supplementary Information Fig. S2 and S3). Chemical elemental analysis by energy dispersive X-ray analysis (EDX) confirmed the spicule composition (see Supplementary Information Fig. S4) attributed to siliceous spicules belonging to the freshwater tree sponge *Drulia uruguayensis (Demospongiae*, Porifera) (see Supplementary Information Fig. S5). Synchrotron phase-contrast enhanced μ-CT analysis of potsherds was able to differentiate the spicules from the surrounding clay. Edge enhancement (due to inline Fresnel propagation recorded in the X-ray radiographs) showed the spicule boundaries through phase-contrast effects revealing the co-alignment of spicules in different regions as clearly seen in Fig. 1d,e. SEM inspection (Fig. 1f) also showed evidences of preferential spicule orientation - an underlying feature found in all analysed potsherds (see Supplementary Information Fig. S3).

We determined the stiffness and extent of crack propagation of size-defined potsherd fragments as a function of the spicule orientation (Fig. 2). SEM inspection revealed that, when spicules were oriented orthogonally to the load, the crack propagates by contouring the spicules (Fig. 2b) following a non-linear path. After structural failure, the spicules embedded in the ceramic matrix retain their integrity (Fig. 2c). We determined a Young’s modulus of 794 ± 19 MPa (Fig. 2d).

In contrast, when a load was applied parallel to the orientation of the spicules the crack propagated between the spicules and along the spicule axes (see Supplementary Information Fig. S6). Although the potter’s intention to add the siliceous spicules remains largely elusive^22^, our results show that presence of oriented siliceous spicules play an important role for improving stiffness of the ceramics and inferring a possible improvement of fracture toughness^23^,^24^,^25^.

We studied the mechanical properties of individual siliceous spicules *S. domuncula* (*Demospongiae*, Porifera). These consist of straight or slightly curved siliceous spicules (tylostyles) with lengths spanning between 100 and 500 μm and diameters between 5 and 10 μm (20) (Fig. 3a). We determined the organic fraction in individual siliceous spicules by nanothermogravimetric analysis (nano-TGA)^26^. The weight of several spicules was calculated by measuring the difference between the resonance frequencies of unloaded (ƒ_1_) and loaded (ƒ_2_) cantilevers yielding values of 9–10 ng (see Supplementary Information Fig. S7 and S8). One representative example with a weight of 9.7 ng is shown (see Supplementary Information Fig. S9d step 2). The spicule was placed onto the cantilever and subsequently heated incrementally in: a) 120 °C, 30 min and b) 500 °C, 1 h. (see Supplementary Information Fig. S8d step 3 and 4). By measuring the differences between the resonance frequencies (ƒ_2_, ƒ_3_) (see Supplementary Information Fig. S7) at room temperature, water (step a) and organic content (step b) were determined to be 1.5 ± 0.2% and 19 ± 3% wt, respectively (see Supplementary Information Fig. S8d). No morphological changes were observed after repeating heating steps (see Supplementary Information Fig. S8e). FT-IR ATR analysis of natural spicules (see Supplementary Information Fig. S8f, black line) and heat-treated spicules (120 °C, 30 min and 500 °C, 1 h) (see Supplementary Information Fig. S8f, red and green lines) showed that IR absorptions of the amide I band (1600–1700 cm^−1^ region) disappeared after heating the sample at 500 °C, 1 h (see Supplementary Information inset Fig. S8f)^27^ confirming the absence of organic material.

The mechanical properties of isolated siliceous spicules were determined by fixing them onto the edge of a silicon wafer with epoxy glue. A tipless AFM cantilever was used to measure the force at different positions from the base of the single suspended spicule (Fig. 3b,c). From the stiffness and deflection of the spicule at different loading positions, we calculated the Young’s modulus of the material. Before mechanically testing individual spicules, their radii were determined by cutting cross-sections using a focused ion beam (FIB) and imaging with scanning electron microscopy (see Supplementary Information Fig. S9). SEM images showed an inner hollow part with a radius (*r*_2_) much smaller than the outer radius (*r*_1_). To analyse our mechanical testing measurements, we assumed a hollow cylindrical geometry with constant outer and inner radii. The force *F* required to deflect a hollow cylinder by a distance (δ) applied at a distance *x* from the base is given by eq. (1):


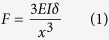


Here, 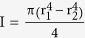 is the area moment of inertia. The stiffness (k = F/δ) was determined from a plot versus the loading position (*x*) for individual spicules according to eq. (2):


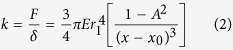


using 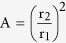, the offset (*x*_0_), and the Young’s modulus *E* as fitting parameters, while leaving *r*_1_ fixed. From fits of several experiments, a Young’s modulus of 28 ± 5 GPa was obtained for natural spicules (before heating) (Fig. 3c) and 46 ± 8 GPa for spicules after heating at 500 °C/1 h, where *r*_1_ values between 2.1 and 3.8 μm were used (Fig. 3d). For commercial glass fibers with a radius of 6.3 μm, we obtained a Young’s modulus of 44 ± 5 GPa, similar to spicules after heating at 500 °C (see Supplementary Information Fig. S10). For other non-heated treated siliceous sponge spicules^17^,^18^ Young’s moduli of 14 GPa^28^ and 68 GPa^29^ have been reported. The fracture properties and ultimate strength of immobilized siliceous spicules were examined using SEM with a micromanipulator. Both spicules states (before and after heating at 500 °C/1 h) displayed excellent bending properties (Fig. 3e,f and movie S1 and S2, Supplementary Information) even after several bending cycles (see Supplementary Information Fig. S11 and S12). Assuming a linear stress-strain curve up to the observed fracture strains of 5–7% and using the measured Young’s modulus for the spicules before and after heat treatment, we calculated a maximum stress of *σ* = 2.0 ± 0.4 GPa and *σ* = 2.4 ± 0.4 GPa right before fracture (see Supplementary Information Fig. S13). The series in Fig. 3f and S13 shows an extreme case, where right before breakage, the spicule flipped into an out of plane bending. For the calculation, we actually used the bending radius from Fig. 3f,iii, which could underestimate the real maximum value observed. SEM analysis of both types of spicules showed a typical glassy conchoidal fracture (Fig. 3e). Values of strength in the range of several GPa for siliceous materials can only be expected if the surfaces are almost defect free^30^.

We set out to study the effects of (i) firing temperature, (ii) incorporation of different additives (sand, glass fibres and siliceous spicules, 1 and 5% wt) and (iii) spicule orientation on the stiffness and crack propagation of clay composites rods (10 × 4 mm) (see Supplementary Information Fig. S14 for clay chemical composition and Fig. 4a). We fabricated these by using the pre-colonial ceramic roll-coil technique (also called by “*rolete*” or “*acordelata*”) learned *in situ* from Amazonian natives (see Supplementary Information Fig. S15). After firing the rods (no additives) at 400 °C, 500 °C and 600 °C for 1 h, we observed an increase of the rod shrinkage (21.7, 24.1 and 26.4%) and of the Young’s modulus (348 ± 0.02, 425 ± 2.2, 451 ± 1.5 MPa) (see Supplementary Information Fig. S16 for representative stress-strain curves). For all further experiments, the clay rods were heat treated at 500 °C for 1 h. Ceramic rods prepared with siliceous spicules (1 and 5% wt) showed a reduced shrinkage (17.5 and 11.5%), but an unexpected and significant, almost 2-fold, of the Young’s modulus (724 ± 3.2 and 836 ± 3.1 MPa) that perfectly correlates with the Young’s modulus determined for the Amazonian potsherd (794 ± 19 MPa). In contrast, addition of commercially available glass fibers (1 and 5% wt) reduced the shrinkage (17.5 and 11.0%) and maintained or reduced the Young’s modulus (439 ± 2.1 and 213 ± 1.4 MPa) as compared to pure clay. Incorporation of sand (1 and 5% wt) has a negligible effect on shrinkage (22.1 and 23.6%) and lead to a Young’s modulus decrease (489 ± 1.7 and 369 ± 1.5 MPa).

Finally, we assessed the effect of siliceous spicule orientation on the stiffness and crack propagation of the ceramic rods. Interestingly, we found that the application of the roll-coil technique generated orientation due to shear forces (Fig. 4b left image) whereas kneading results in randomly distributed spicules (Fig. 4b right image). Under otherwise identical experimental conditions, we observed a decrease of the Young’s modulus (698 ± 2.2 and 450 ± 3.1 MPa) for clay rods containing randomly distributed spicules (1 and 5% wt), respectively, where the latter is close to the Young’s modulus of the ceramic rods without additives (425 ± 2.2 MPa). Figure 4c,i,ii shows that randomly distributed spicules (5% wt) have no effect on crack arrest as it propagates linearly throughout the rod. Oppositely, the rods containing highly orientated spicules (5% wt) showed an opposed behaviour, i.e., the siliceous spicules helped to arrest crack propagation (Fig. 4d,i,ii,e) as more energy is required to overcome these micro-inclusions, particularly, when positioned orthogonally.

It is well established that the improvement of ceramics’s fracture toughness relies on the incorporation of a variety of reinforcing phases (e.g. carbon nanotubes, glass fibres, whiskers) within a matrix (e.g. concrete, advanced ceramics)^25^. Factors such as chemical and structural nature, size (micro, nano) and mechanical properties of the reinforcing phases, their interaction and organization in the matrix play a crucial role on the design of composite materials^23^,^24^. Despite the fact that all those factors were obviously unknown to pre-colonial Amazonian civilizations, their empirical technological knowledge was a product of experimentation and interrelation of humans with their biopsychological aspects, environment (both social and natural) and their artefacts^31^. Moreover, it is interesting to find an extensive archaeological record of composite materials that represent a unique event in the history of mankind - the use of locally available biological systems (fresh water sponges) and their skeletal elements (siliceous spicules) as highly functional reinforcing phases.

Mechanical studies on the reinforcing elements (*Demospongiae’s* siliceous spicules) and the correlation with their role in pre-colonial Amazonian ceramic composites was lacking and, thus, raising speculation among archaeologists and anthropologists over its real function and potter’s intentional use during the manufacturing process^6^,^12^,^13^. To shed light on this fundamental question, we have used a stepwise approach. As a first step, we exploited the mechanical properties of isolated siliceous spicules from *Demospongiae* using our previous knowledge from similar microsized systems^26^. As a second step, we have fabricated ceramic composite models using traditional Amazonian techniques (roll-coil) using different additives (sand, glass fibres and siliceous spicules) and correlate their content with stiffness. We observed that isolated siliceous spicules behave almost defect free glass fibres retaining their mechanical strength even after firing at 500 °C (absence of organic molecules). When combined with clay (in the form of ceramics), prevent overall structural shrinkage and yield the highest stiffness (724 ± 3.2 and 836 ± 3.1 MPa for 1 and 5% wt) among all additives used. Microscopic observations of our ceramic models showed that shearing forces intrinsic to the continuous application of the roll-coil technique generate alignment/orientation of reinforcing phases (glass fibres and siliceous spicules). The similarity between our models and the potsherds are striking (Fig. 1f, Fig. 4b and see Supplementary Information Fig. S2). Comparative analysis between ceramic models with oriented and randomly distributed spicules show that randomly distributed spicules have no contribution to the overall structural improvement, highlighting the importance of spicule orientation in both stiffness and crack arresting of pre-colonial South-America ceramics (Fig. 4f). Composites have been known for several thousands of years as an expression of the will and fascination to combine existing materials in such a way that new properties could emerge. Pre-colonial South-America ceramics are no exception here. Exceptional and unique is the fact that they used locally available biological systems and their skeletal elements - which retain their mechanical properties after firing - to reinforce an abundant material with low mechanical properties and limited applications. This technological leap - from restricted to almost endless applications – have had a profound impact on pre-colonial South-American civilization development. Until today this technology still remains valid.

## Methods

### Spicule characterization

Spicules were kindly provided by Dr. Renato Batel (Ruđer Bošković Institute, Rovinj, Croatia) and cleaned from the tissue by using a previously described procedure^16^. Infrared analysis was performed on a Nicolet Nexus spectrometer fitted with a Golden Gate attenuated total reflection (ATR) accessory (Thermo Nicolet). Spectra were recorded at 4 cm^−1^ resolution, averaging 32 scans^27^. *S. domuncula* spicules were glued to the edge of a silicon wafer using a two-component epoxy resin (UHU endfest 300) under an optical microscope coupled to 3-axis oil micromanipulator (Narishige MMO, Tokyo, Japan). Mechanical and nano-TGA of siliceous spicules were determined as previously described^26^. A detailed description can be found by Supplementary Information.

### Preparation of ceramic composite rods

Clay was obtained from a natural source in Portugal and its composition determined by elemental analysis (see Supplementary Information Fig. S15). A commercially available aluminium tube was cut into small pieces of standardized sizes (10 mm length and 4 mm internal diameter) that were used as moulds where the clay was filled. In all cases, the clay-aluminium rods were placed in a horizontal tube furnace. Afterwards, the samples were allowed to cool to room temperature. The cylindrical rods were carefully removed from the aluminium moulds, the height and diameter measured and assayed for the mechanical properties. In the following set of experiments, three samples for each settings/conditions/additives were fabricated. **a) Temperature/firing:** clay without additives were heated at 400, 500 and 600 °C with a heating rate of 2 °C/min with a stationary phase at the corresponding temperature for 60 min. **b) Additives:** Clay was mixed with following additives: sand (Clean Sand #4, Fluka, Germany), chopped strands of E-glass glass with a nominal diameter of 13 μm and a nominal length of 100 μm (chopped strands for thermoplastic reinforcement, OCV^TM^ Reinforcements Owens Corning) and *S. domuncula* spicules with 1 and 5% wt. We used the roll-coil technique for E-glass fibres and *S. domuncula* spicules the roll-coil technique as described previously^13^ which we learned *in situ* with Amazonian native potter’s during our Amazonian Expedition (February 2014) (see Supplementary Information Fig. S16 online). The samples were placed in a furnace at 500 °C for 60 min using a heating rate of 2 °C/min. **c) Orientation:** Clay was kneaded with NaOCl-cleaned *S. domuncula* spicules (1 and 5% wt). The samples were placed in a furnace at 500 °C for 60 min and a heating rate of 2 °C/min.

### Collection of samples

The analysed ceramic samples were collected in controlled excavations at five archaeological sites located on the central Amazonian region/Urubu river (Itacoatiara and Silves, Amazonas, Brazil), by a team of archaeologists led by Dr. Helena Lima. Evidences of indigenous sedentary large settlements in this area date back to the 5^th^ century AD (*anno domini*) 1,490 ± 40 B.P. (years before present) and extend to the contact and colonial period (see Supplementary Information Fig. S1). A governmental permission was issued to permit its international translocation and analysis (chemical and mechanical). All samples were labelled according to the following system: North (N)/East (E) coordinate that refers to the exact location of the ceramic collection at the archaeological site, according to an arbitrary Cartesian grid established for each site. Thus, N1049/E880 refers to the position of the sample.

### Chemical composition and microscopic imaging of potsherd

Longitudinal sections of potsherds were carried out using a scalpel and/or a saw. The pieces were fixed onto a carbon tape (Plano GmbH, Wetzlar, Germany) and it morphology analysed under a scanning electron microscope (SEM) (JEOL JSM-6710F, JEOL Germany GmbH, Germany) at 2 kV and within a vacuum chamber at 2.5 × 10^−6^ mbar. Chemical elemental analysis of potsherds, was carried out using a X-flash detector 4010 (Brucker AXS Microanalysis GmbH, Germany) using 15 keV of acceleration. The spectra (mapping) were acquired, recorded and analysed using Esprit software 1.8 (Brucker AXS Microanalysis GmbH, Germany).

### Determination of mechanical properties

The compression tests of both pre-colonial amazonian potsherd cuts (with defined dimensions of 20 × 20 × 10 mm) and ceramic rod models (10 × 4 mm) were performed using a Zwick universal testing machine (Z010, Zwick/Roell, Zwick GmbH, Ulm, Germany) at room temperature, equipped with a 10 kN-load cell under the following conditions: preload of 0.2 N/10 mm/min and testing speed/loading rate of 0.5 mm/min. The force (N)-displacement curves (mm) were recorded using testXpert II software (*Zwick* GmbH & Co. KG, Ulm, Germany). Stress-strain curves and respective Young’s modulus were calculated using Origin Pro v8.0725 (OriginLab Corporation, Northhampton, USA) linearly fitting the elastic section of each independent measurement. All samples were measured in triplicate.

### Synchrotron microtomography

Phase contrast-enhanced imaging was performed on BAMline of the BESSY-II synchrotron storage ring of the HZB (Helmholtz-Zentrum Berlin, Germany). The samples were imaged using energy of 28 keV, employing 1,500 projections with an 80 mm sample-to detector distance, and an effective pixel size of 4.35 μm^32^. Data was normalized using a custom-written code and then reconstructed by filtered backprojection using the free version of NRecon (NRecon v. 1.6.8, BrukerCT, Belgium). Data manipulation and visualization was performed using ImageJ^33^ and CTvox (v. 2.4, BrukerCT, Kontich, Belgium).

## Additional Information

**How to cite this article**: Natalio, F. *et al.* Siliceous spicules enhance fracture-resistance and stiffness of pre-colonial Amazonian ceramics. *Sci. Rep.* **5**, 13303; doi: 10.1038/srep13303 (2015).

## Supplementary Material

Supplementary Information

## Figures and Tables

**Figure 1:**
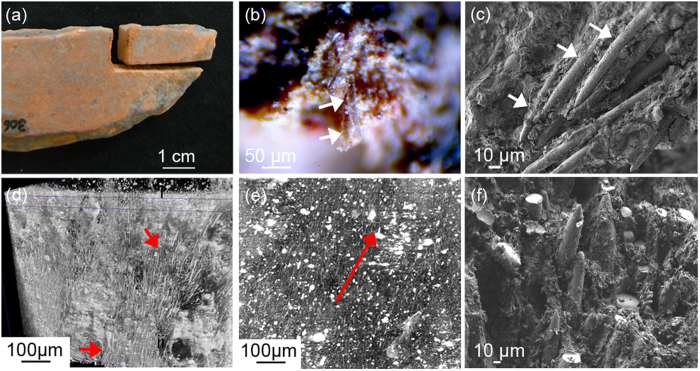
Pre-colonial Amazonian potsherds. (**a**) Digital image of representative ancient pre-colonial Amazonian potsherd (vase wall, Santa Helena archeological site, N1049 E880, Brazil). (**b**) Low magnification optical microscopy image showing the presence of glassy structures attributed to siliceous spicules of the freshwater tree sponge Demospongiae *Drulia uruguayensis* (cauíxi). (**c**) SEM inspection of the same potsherd showing rod-like structures inside the clay matrix and displaying a preferential orientation. (**d**) Cross-section in the phase contrast-enhanced μ-tomography 3D data reveals co-alignment of spicules in different regions within the intact bulk of the sample. (**e**) Averaged image from (**d**). In both cases the spicules show preferential orientation. (**f**) SEM image of potsherd showing spicule alignment within the ceramic matrix.

**Figure 2:**
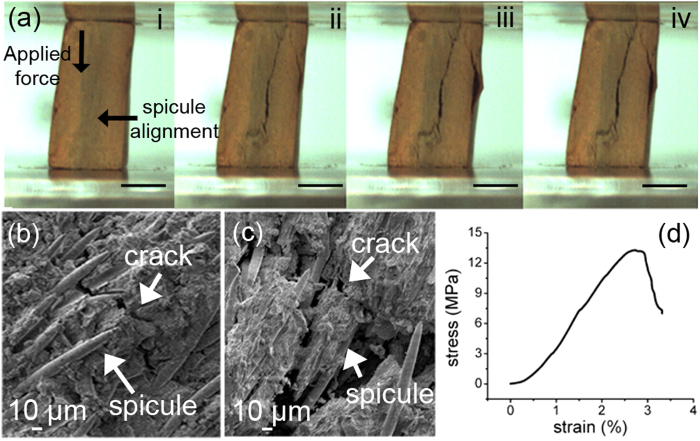
Mechanics of pre-colonial Amazonian potsherds. (**a**) Sequence of digital images showing the mechanical behaviour of a potsherd (Santa Helena archaeological site, N1049 E880, Brazil) under compressive load orthogonally applied to spicule orientation. (**b**) SEM inspection at initial crack propagation. (**c**) SEM after structural failure. The crack surrounds the siliceous spicules, which remain embedded and structurally undamaged in the clay. (**d**) Representative stress-strain curve (1 out of 3 measurements) of pre-colonial Amazonian potsherd.

**Figure 3:**
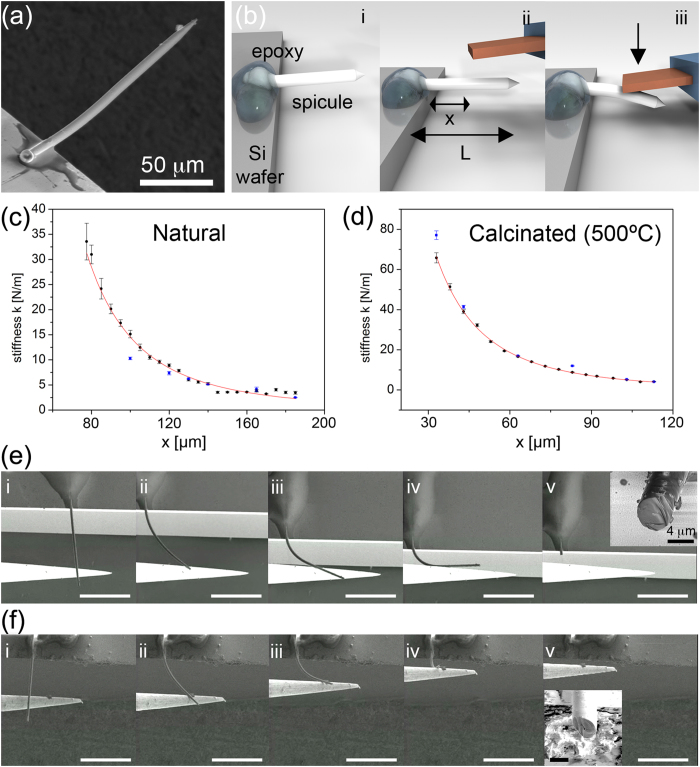
Mechanical properties of *Demospongiae* spicules. (**a**) SEM image of an immobilized spicule fixed at the edge of a Si wafer with epoxy glue. (**b**) The flexural response of a fixed siliceous spicule of length *L* to a well-defined load applied at different positions *x* along its main axis was probed using a tipless AFM cantilever. From the linear relationship between applied force and deformation, the position dependent stiffness *k* of the clamped spicule was obtained. (**c**,**d**), Measured values of *k*_*S*_ versus *x/L* and the fit (red dashed) for the natural (**b**) and the heat-treated spicule (500 °C for 1 h) (**d**). (**e**,**f**), The fracture properties of silica spicules were probed using a micromanipulator and recorded *in-situ* in an SEM. White scale bars are all 100 μm. (**f**) for natural (*i-v*) and (**e**) after heat treatment (500 °C/1 h) (*i-v*). *Inset:* SEM images (e, v and f, v) showing a glassy conchoidal fracture. Scale bar: 10 μm.

**Figure 4:**
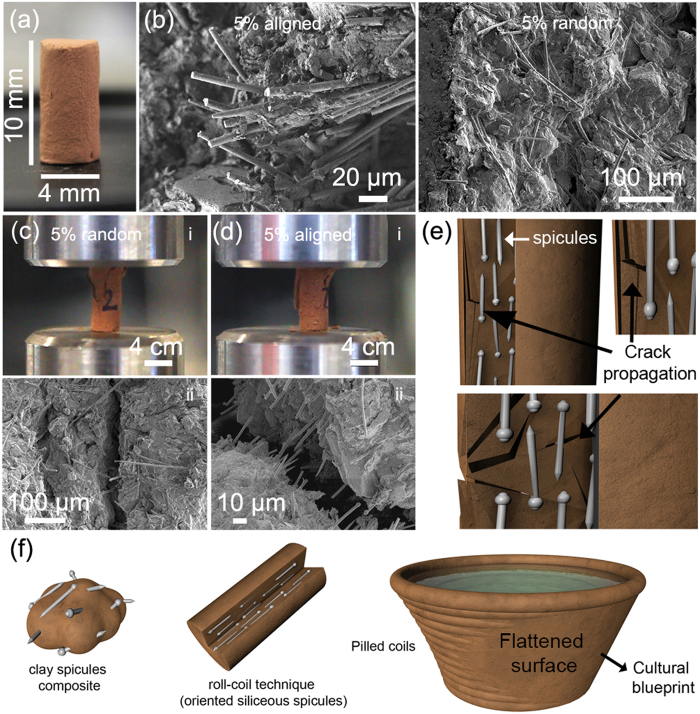
Orientation vs. mechanical properties. (**a**) Digital camera image of a standardized clay rod (10 × 4 mm) after heat treatment (500 °C for 1 h). (**b**) Scanning Electron Microscope (SEM) of clay rods containing siliceous spicules (5% wt) fabricated by the coil-roll technique (left image) and kneaded (right image) show a clear orientation and random distribution of the spicules, respectively. (**c**), *i,* Mechanical behavior of clay rods with randomly distributed spicules (5% wt). *ii,* SEM inspection after structural failure shows that spicules do not prevent crack propagation. (**d**) *i,* Mechanical behavior of clay rods with highly orientated spicules (5% wt). *ii,* similar behavior as for potsherds after structural failure, i.e., the crack propagates by surrounding the aligned spicules. The spicules remain structurally undamaged. (**e**) Schematic representation of the role of siliceous spicules in preventing crack propagation. (**f**) Schematic representation of the ancient Amazonian coil-roll technique used to orient the siliceous spicules (*Demospongiae*) to enhance the mechanical properties of pottery (e.g. utilitarian’s, cultural expression items).
