# Supplementary material for: Siliceous spicules enhance fracture-resistance and stiffness of pre-colonial Amazonian ceramics
Source: Sci Rep. 2015 Aug 27;5:13303. doi: 10.1038/srep13303 (PMC4550886; doi:10.1038/srep13303)
Supplement: Supplementary Information [file srep13303-s1.doc]

**Supporting Online Material**

**Siliceous spicules enhance fracture-resistance and stiffness of pre-colonial Amazonian ceramics**

Filipe Natalio^1,2*^, Tomas P. Corrales^3,4^, Stephanie Wanka^3^, Paul Zaslansky^5^, Michael Kappl^3^, Helena Pinto Lima^6^, Hans-Jürgen Butt^3^ and Wolfgang Tremel^1*^

^1^ Institut für Anorganische Chemie und Analytische Chemie, Johannes Gutenberg-Universität Duesbergweg 10-14, 55099 Mainz, Germany

^2^ Institut für Chemie, Martin Luther Universität Halle-Wittenberg, Kurt-Mothes Str. 2, 06120 Halle (Saale) Germany.

^3^ Max Planck-Institut für Polymerforschung, Ackermannweg 10, 55128 Mainz, Germany

^4^ Instituto de Alta Investigación, Universidad de Tarapacá, Casilla 7-D Arica, Chile

^5^ Julius Wolff Institut, Charité - Universitätsmedizin Berlin, Augustenburger Platz 1, 13353 Berlin, Germany

^6^ Museu Paraense Emílio Goeldi (MPEG), Av. Perimetral 1901, Terra Firme, 66070-530, Belém, Brasil

**Methods**

***Demonspongiae* spicules.** Spicules were kindly provided by Dr. Renato Batel (Rudjer Boskovic Institute, Croatia) and cleaned from the tissue by using a previously described procedure^1-3^. Briefly, 1mm^3^ of frozen *S. domuncula* was immersed in a solution of an aqueous solution of NaOCl (5%) for 1d at 4°C. The white precipitate was washed three times with distilled water followed by centrifugation (2000xrpm, 10 min at 4°C). The needles were air-dried at room temperature.

**FT-IR ATR.** Infrared analysis was performed on a Nicolet Nexus spectrometer fitted with a Golden Gate attenuated total reflection (ATR) accessory (Thermo Nicolet). Spectra were recorded at 4 cm^-1^ resolution, averaging 32 scans^1^.

**Mechanical testing of single *Demonspongiae* spicules by AFM.** *S. domuncula* spicules were glued to the edge of a silicon wafer using a two-component epoxy resin (UHU endfest 300) under an optical microscope. For this purpose, the spicules were picked up at their end by touching them with an AFM that is maneuvered with a micromanipulator (Narishige MMO, Tokyo, Japan) and placed near the edge of the silicon wafer. Subsequently, the cantilever end was dipped into epoxy glue, and a micrometer-sized glue drop was placed near the edge of the silicon wafer. Next, a fresh cantilever was used to move the deposited spicule over the silicon surface towards the drop of glue. After one end of the spicule was immersed in glue the free end was rotated until most of it was suspended over the edge of the silicon wafer. After this step the second cantilever was dipped in glue and a second bigger drop was placed on the previous one in order to leave the spicule well clamped to form a single-sided clamped beam. The silicon wafer was mounted on the sample stage of the Dimension 3100 AFM (Bruker, Billerica, MA), and a tipless AFM cantilever (µmasch NSC12 tipless, ISB Ltd, Sofia, Bulgaria) was employed to probe the flexural response of the spicule beam by taking force distance curves on defined positions along the main axis of the spicule. From the comparison with force-distance curves taken on the silicon wafer itself, the effective spring stiffness *k* of the spicule was obtained depending on the relative position of the loading point. For each loading point 30 - 40 force-distance curves were recorded. The applied load was kept constant along single spicules (150nN-300 nN). Spring constants of AFM cantilevers were determined using the thermal noise method^4^.

As the length of the spicules exceeded the maximum range of the AFM scanner of 100 µm, the beam deflection vs. position curves were taken in two steps. During the first one, probing started at the free end of the spicule, proceeding towards the clamped end exploiting the full piezo scan range of the AFM. For the second step, we made use of the motorized sample stage to reposition the spicule along its axis by a defined distance to allow full coverage of the remaining part by a second piezo-scan.

We were not able probe the full length of the spicule, because close to the clamping point, a sufficient linear bending of the spicule (δ > 5 nm) was harder to achieve within the linear deflection regime of the AFM cantilever. SEM images of the samples were taken after the AFM measurements in order to determine the length *L* and radius *R* of the spicules. We assumed that the spicules are well represented by a homogeneous hollow cylinder. This assumption implies that the values of the Young’s modulus are representative for the whole composite structure, which might differ from that of the single components. Effective stiffness curves (were fitted using Eq. (2) that contains an additional offset *x*_0_. This additional fit parameter was necessary to adjust for uncertainties in the determination of the clamping point.

**Mechanical testing and cross-sectioning of spicules in a FIB/SEM.** Spicules were fixed at one end to a silicon wafer as described above and introduced into the sample chamber of a FEI 600 Nanolab Focused Ion Beam (FIB) / SEM dual beam instrument (FEI, Hillboro, Oregon) equipped with an Omniprobe micromanipulator (Omniprobe Inc. Dallas, Texas). For determining the spicule radius, immobilized spicules were cut with the Ga^+^ ion beam using beam currents of 500 pA followed by a polishing cut using a beam current of 50 pA and then directly imaged by SEM. For testing the fracture behavior spicules were bent by contacting their end with the tungsten tip of the Omniprobe micromanipulator and deflecting them with a linear movement of the SEM sample stage while recording spicule the deflection by continuous SEM imaging. To calculate the breakage stress we determined the minimum bending radius of the spicules from the last SEM image prior to breakage (Fig. S13). From this bending radius *R* and the spicule radius *r_1_*, one obtains the breakage strain as *r_1_*/*R*. The fracture stress was then calculated by multiplying strain with the Young’s modulus.

**Nanothermogravimetric analysis (nanoTGA).** For this experiment a tipless silicon AFM cantilever (μmasch NSC12 tipless, ISB Ltd, Sofia, Bulgaria) was mounted in a commercial JPK Nanowizard AFM (JPK Instruments, Berlin, Germany). Nominal spring constant and resonance frequency of the cantilevers used for these experiments were 16 N/m and 300 Khz respectively. The glass cantilever holder of the AFM allows high temperature calcination experiments, in which the complete holder, including cantilever and spicule, is placed within a high temperature oven. All cantilever spring constants were first measured using the thermal noise^4^. By fitting the thermal noise spectra of the unloaded cantilever, its spring constant and initial resonance frequency was obtained. Single spicules were loaded on the backside of the cantilever using a 3-axis oil micromanipulator (Narishige MMO, Tokyo, Japan). The cantilever was mounted again in the AFM to measure the resonance frequency when loaded with the spicule. After incubating the holder with cantilever and spicule at 120°C and 500°C, thermal noise spectra of the loaded cantilever were recorded. From the observed shifts in the resonance frequency, the mass of the single spicule and its mass change after each heating cycle can be calculated using the following equations:

*
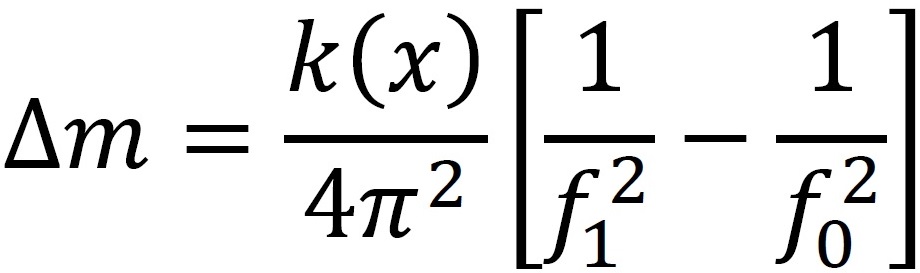
*

where k(x) is the effective spring constant of the cantilever which depends on the position (x) where the spicule is loaded onto it. This position was measured from optical micrographs. This effective spring constant relates to the spring constant at the end of the cantilever (k(L)) that we have determined by the thermal noise method by:

*
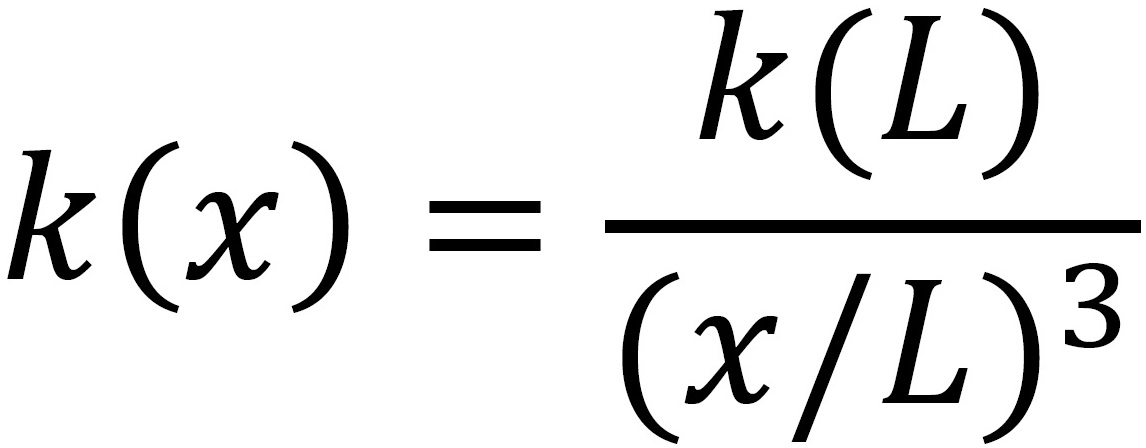
*

Additionally, we accessed the eventual variation of the cantilever resonance frequency upon heating (500ºC for 1h) during three consecutive heating cycles. We observed no changes in the resonance frequency indicating that the heating cycles did not affect the initial resonance frequency and thus did not interfere with the nano-TGA measurements^5^.

**Figures and captions**

**
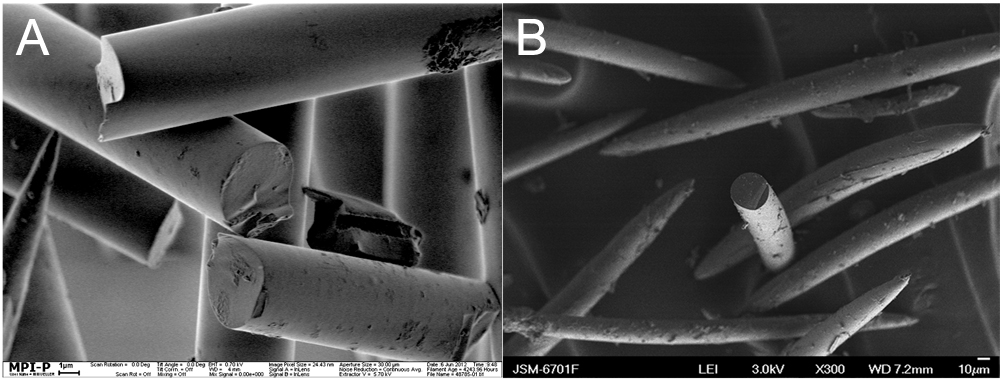
**

**Figure S1.** Scanning Electron Micrographs of broken siliceous spicules isolated from **A**, *Suberites domuncula* and **B**, *Drulia uruguayensis* showing a similar cross-sectional profile, i.e., a dense silica and axial canal.

**
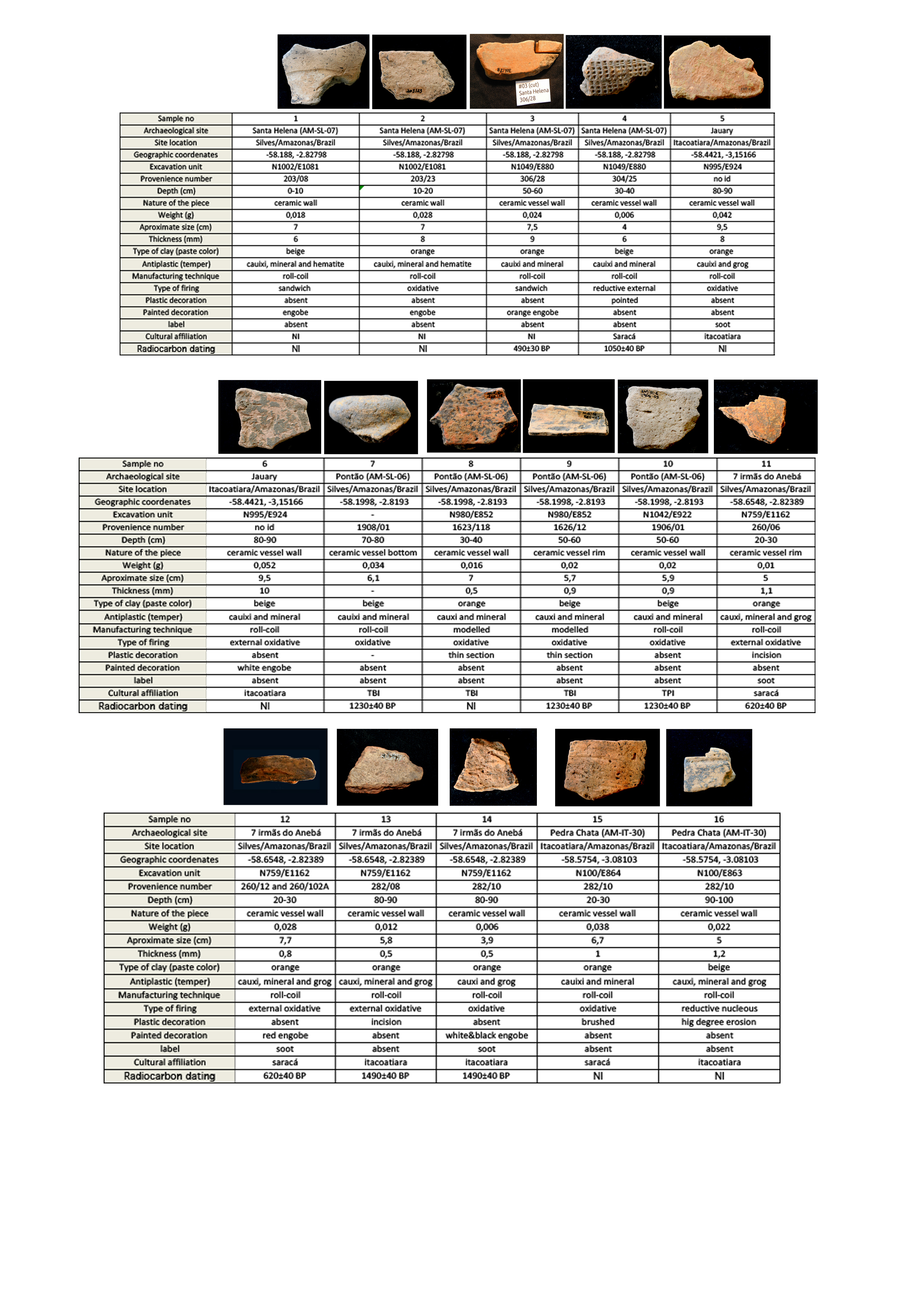
**

**Figure S2.** Comprehensive description of Amazonian pre-colonial archeological potsherds used for analysis.


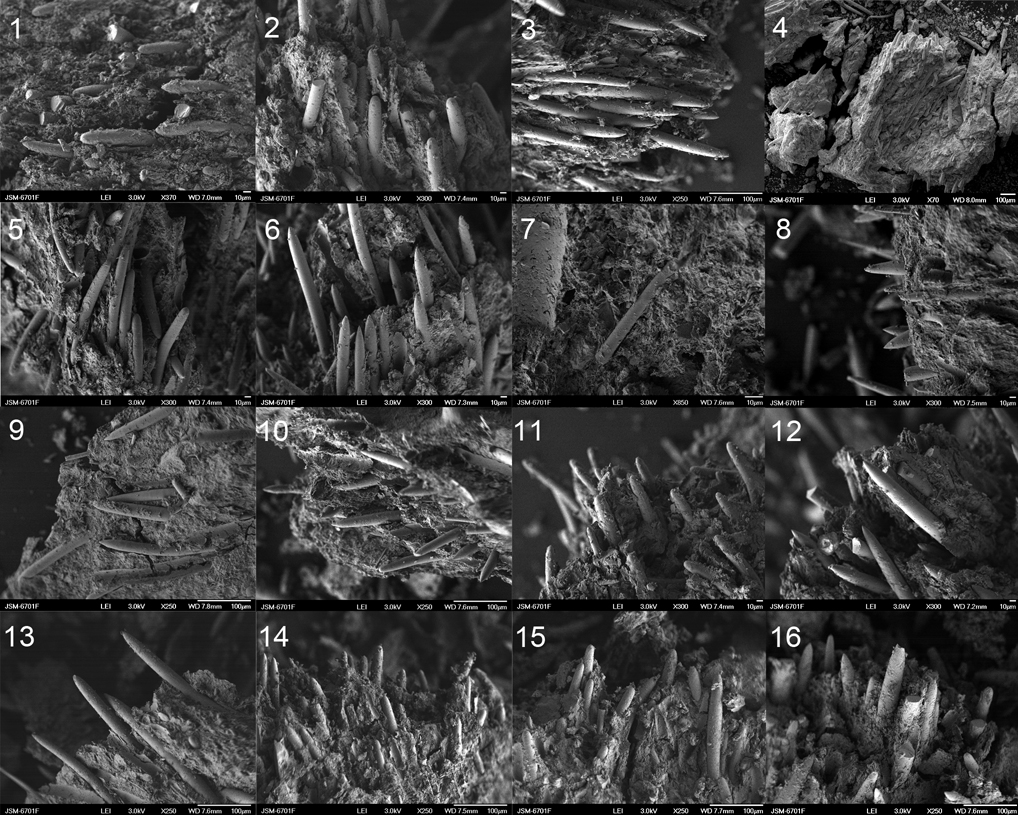


**Figure S3.** Scanning electron microscope images (SEM) of **all** ancient amazonian potsherds showing glassy needle-like structures. In all, preferential orientation of the siliceous spicules within the clay matrix is clearly observed.


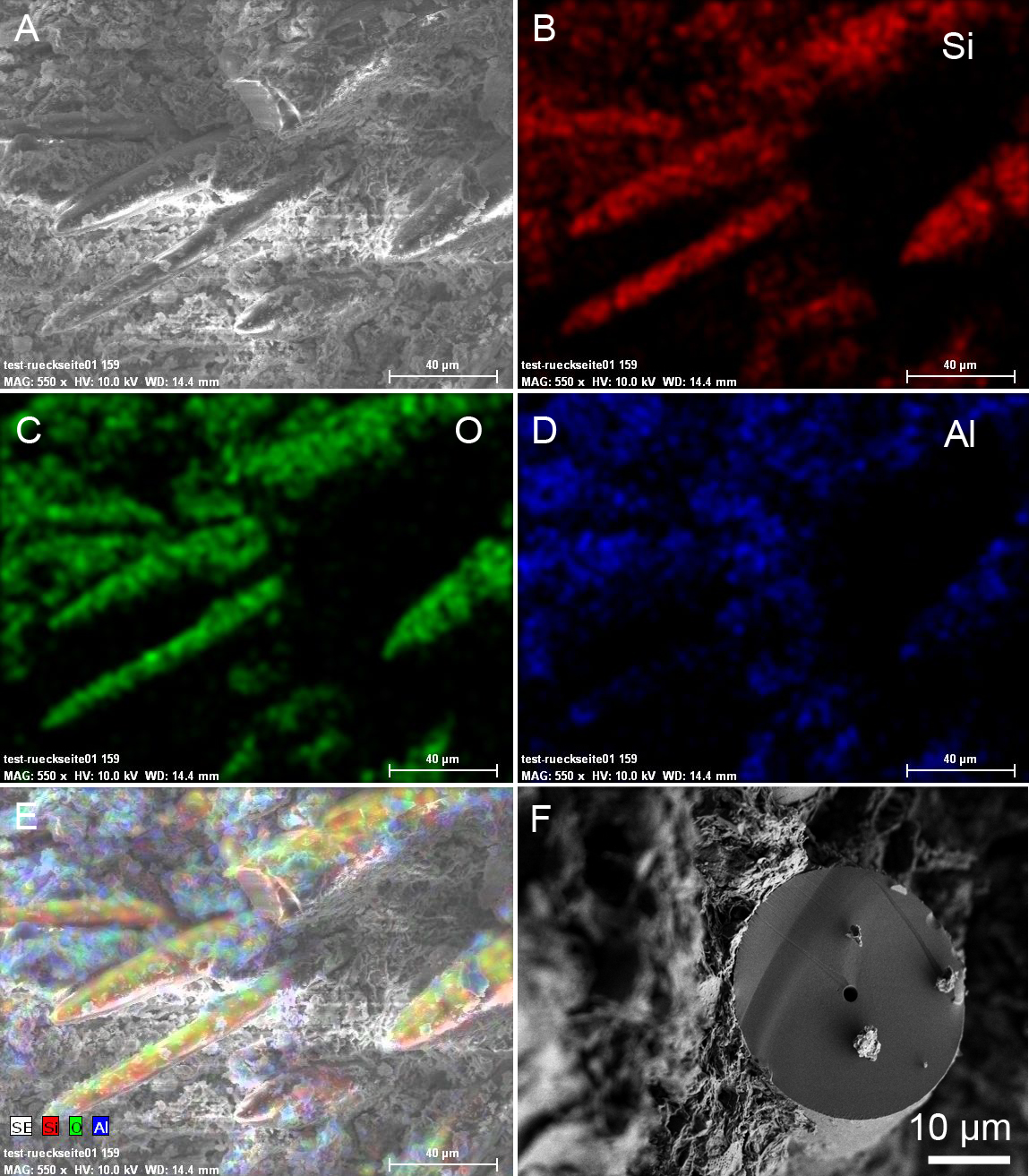


**Figure S4.** Chemical elemental mapping (EDX) and morphological analysis of an Amazonian pottery sherd (Santa Helena archeological site, N1049 E880, Brazil). **A,** SEM image. **B,** Si. **C,** O. **D,** Al. **E,** Overlap. The presence of co-localizing signals of Si and O confirms the siliceous nature of the spicules. **F,** SEM image of spicule inside ceramic matrix displaying the typical *Demospongiea* features. The hollow axial canal denotes the absence of organic material confirming that the sherds underwent heat treatment (firing).

Note: Due to the interferences derived from the clay (aluminium phyllosilicates) we analyse spicules that are partially integrated into the ceramics in order to obtain a clear Si and O signal.


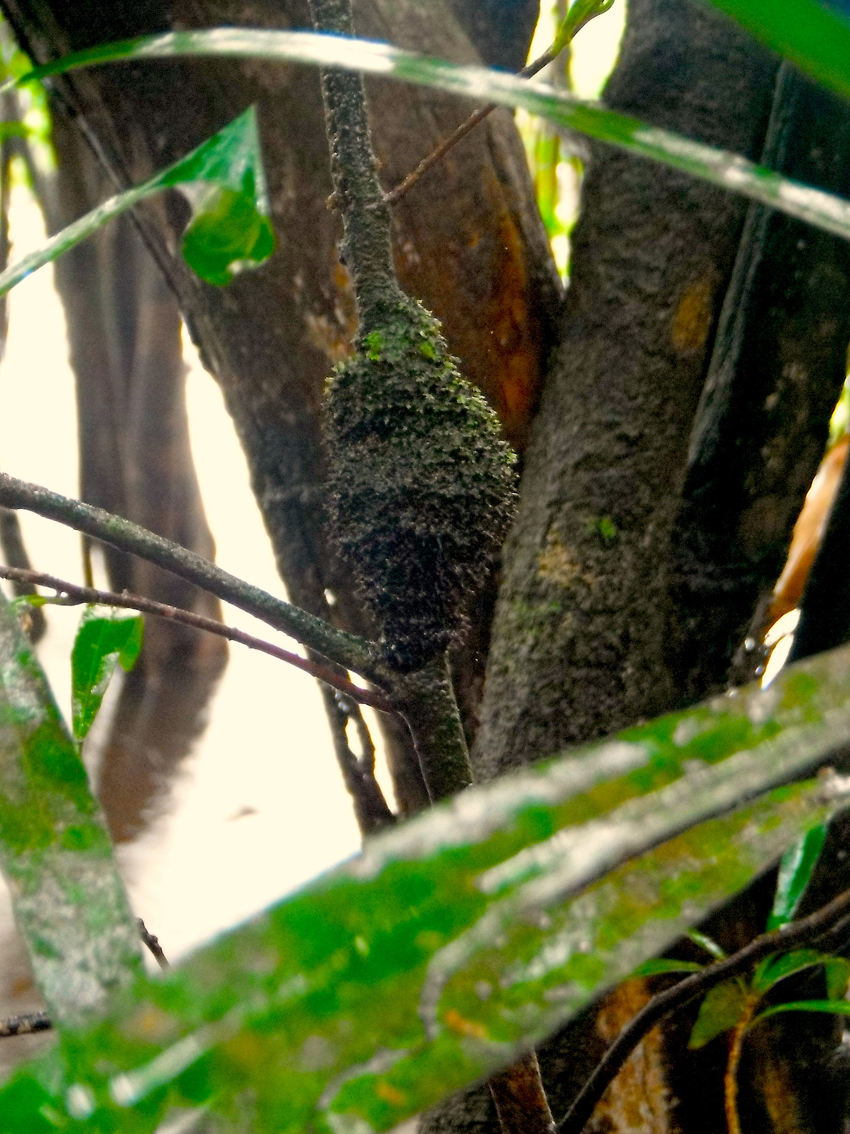


**Figure S5.** Photographic image of a freshwater sponge (cauíxi-tree sponge) from the genus *Drulia* - *Drulia uruguayensis* (*Porifera, Demospongiae, Metaniidae, Drulia*) in its natural enviroment (Amazonia, river Negro).


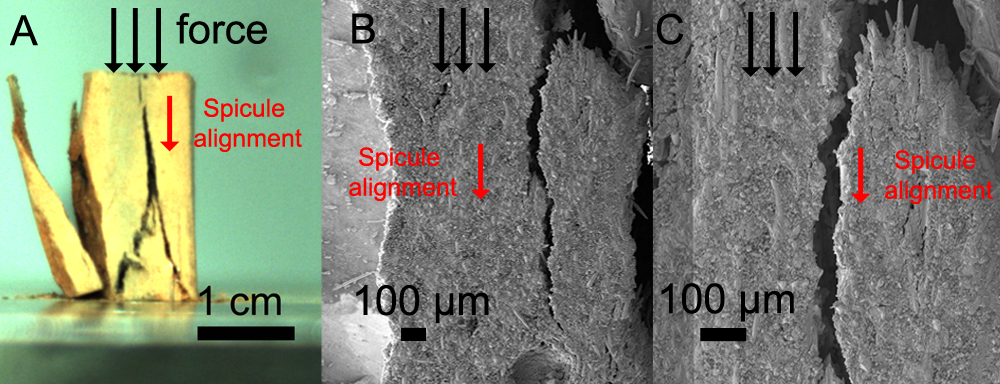


**Figure S6.** Mechanical behavior of a pre-colonial Amazonian potsherd (Santa Helena archeological site, N1049 E880, Brazil) as function of spicule’s preferential orientation. The spicules are orientated in parallel to the compressive load leading to crack propagation and complete structural failure. **A**, Digital camera image. **B**, SEM image. **C**, SEM image at higher magnification.


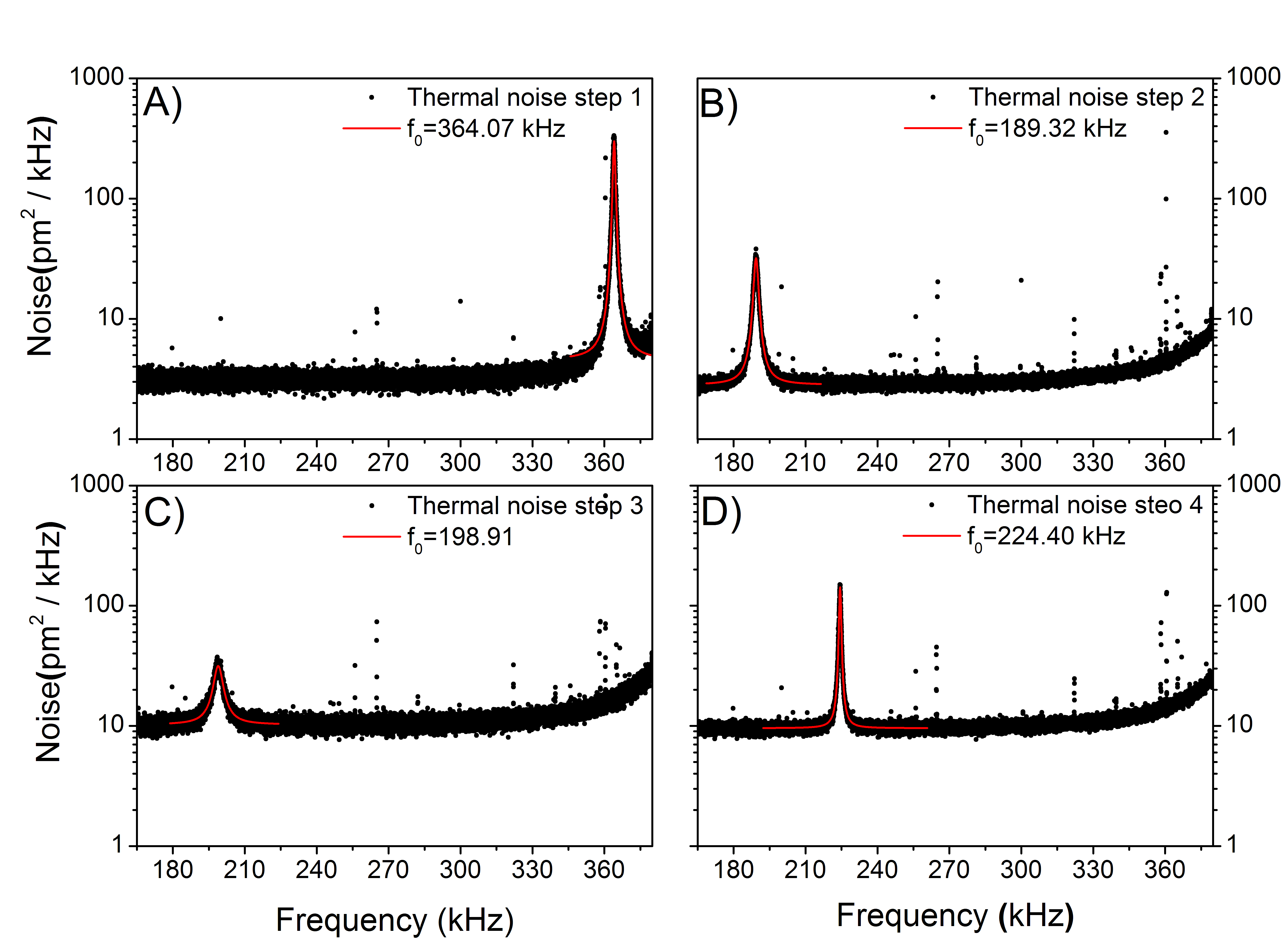


**Figure S7.** **A,** Thermal noise spectrum of a bare cantilever. From a fit of this spectrum the resonance frequency (ƒ_0_= 364 kHz) and spring constant were obtained (*k*= 7 N/m). **B,** After loading this cantilever with a spicule, the thermal noise spectrum yields a lower resonance frequency due to the added mass (ƒ_1_ = 189 kHz). **C,** Heating the holder with cantilever and spicule at 120°C for 1h leads to a mass loss reflected by an increase in resonance frequency (ƒ_2_= 198 kHz). **D,** Finally the spicule is calcinated at 500°C for 1 h, leading to a further mass loss attributed to its organic content, reflected by a further increase in resonance frequency (ƒ_3_= 224 kHz).


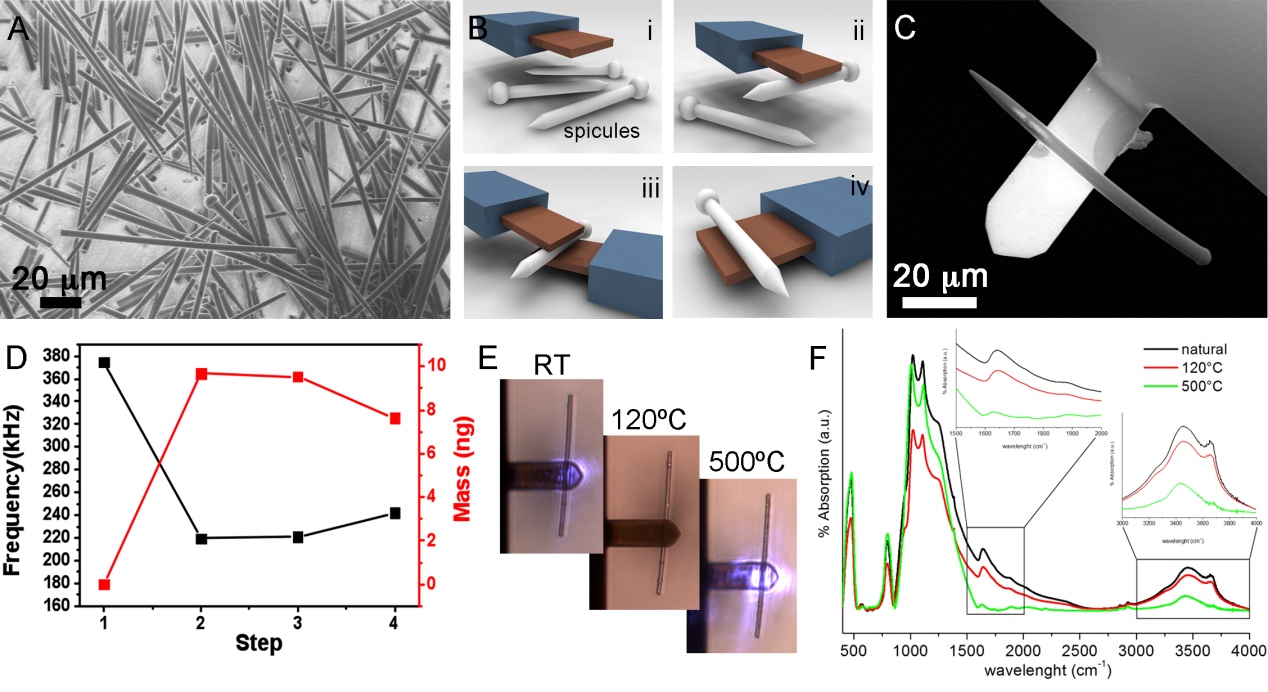


**Figure S8.** **A** SEM image of *S.domuncula* cleaned spicules (tylostyles) showing spicules of different lengths. **B** Sequential procedure of loading *single* siliceous spicules onto an AFM cantilever. A *single* spicule is taken up using a tipless AFM cantilever and loaded onto another AFM cantilever. **C** SEM image of a single spicule loaded onto an AFM cantilever. **D** Nano-TGA. A siliceous spicule was loaded at step 2. Step 3 and 4 shows the change in resonance frequency after heat treatment at 120ºC and 500°C respectively. From step 3 and 4, we infer the amounts to water and organic content to be 1.5 and 19.4% wt, respectively. **E** Sequential images of the spicules loaded onto the AFM cantilever at different stages: initial, after 120ºC (1h) and after 500ºC (1h). No morphological changes were observed. **F,** FTIR-ATR spectrum of naturally occurring spicules (no treatment) (black line), after heat treatment at 120ºC (red line) and at 500ºC (green line) showing the complete absence of organic material (amide I bond region) after 500ºC treatment (inset).


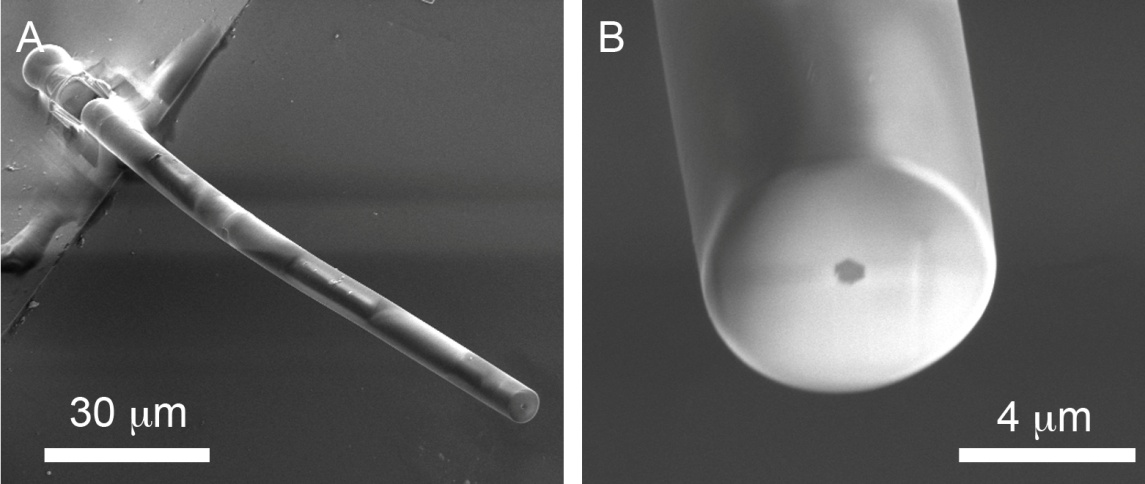


**Figure S9. A,** Scanning electron microscopy images of a *S. domuncula* spicule after FIB cutting at its tip and base. We used this information of determining the spicule inner and outer radius and subsequently the Young’s modulus. The FIB was used to eliminate the sharp tip typically found in *Demospongiae* spicules. For Young’s modulus (E) calculations, we assumed that the spicules are hollow cylinders with constant outer and inner radius. **B**, Higher magnification SEM image of the spicule tip.


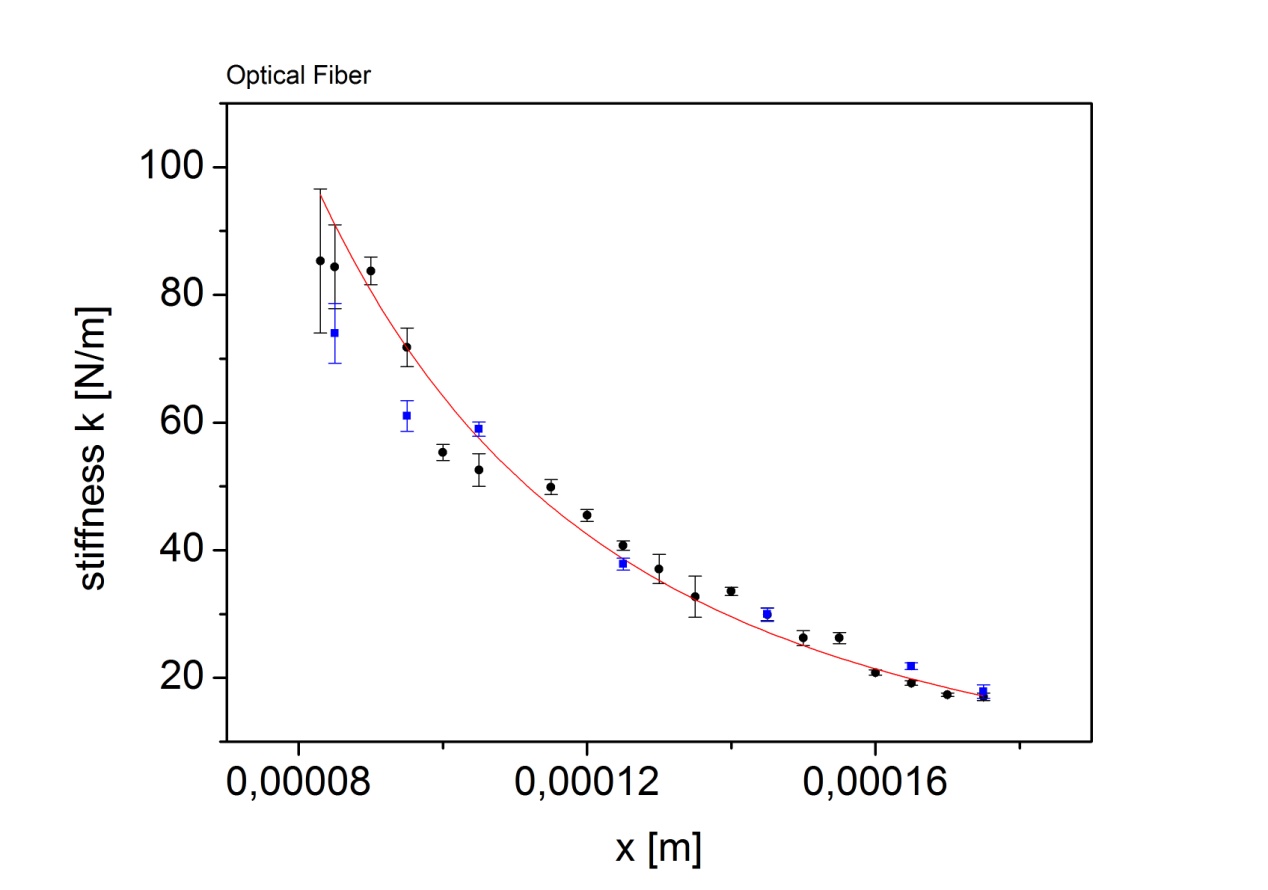


**Figure S10.** Fit of the stiffness versus loading point curve for a commercially available glass fiber. Assuming a constant radius of 6.3 µm, we determined a Young’s modulus of 44 ± 5 GPa.


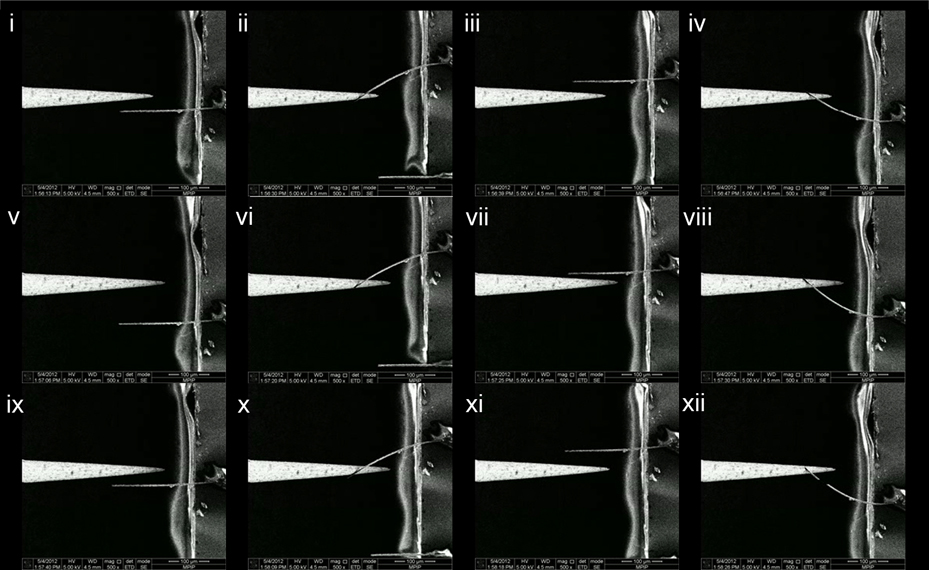


**Figure S11.** Sequence of images showing several bending cycles of immobilized *S. domuncula* spicules (*Demospongiae*). It can be observed that after several cycles, the spicule retains its extreme bending properties and returns to original position. There are no signs of plastic deformation or structural fatigue.


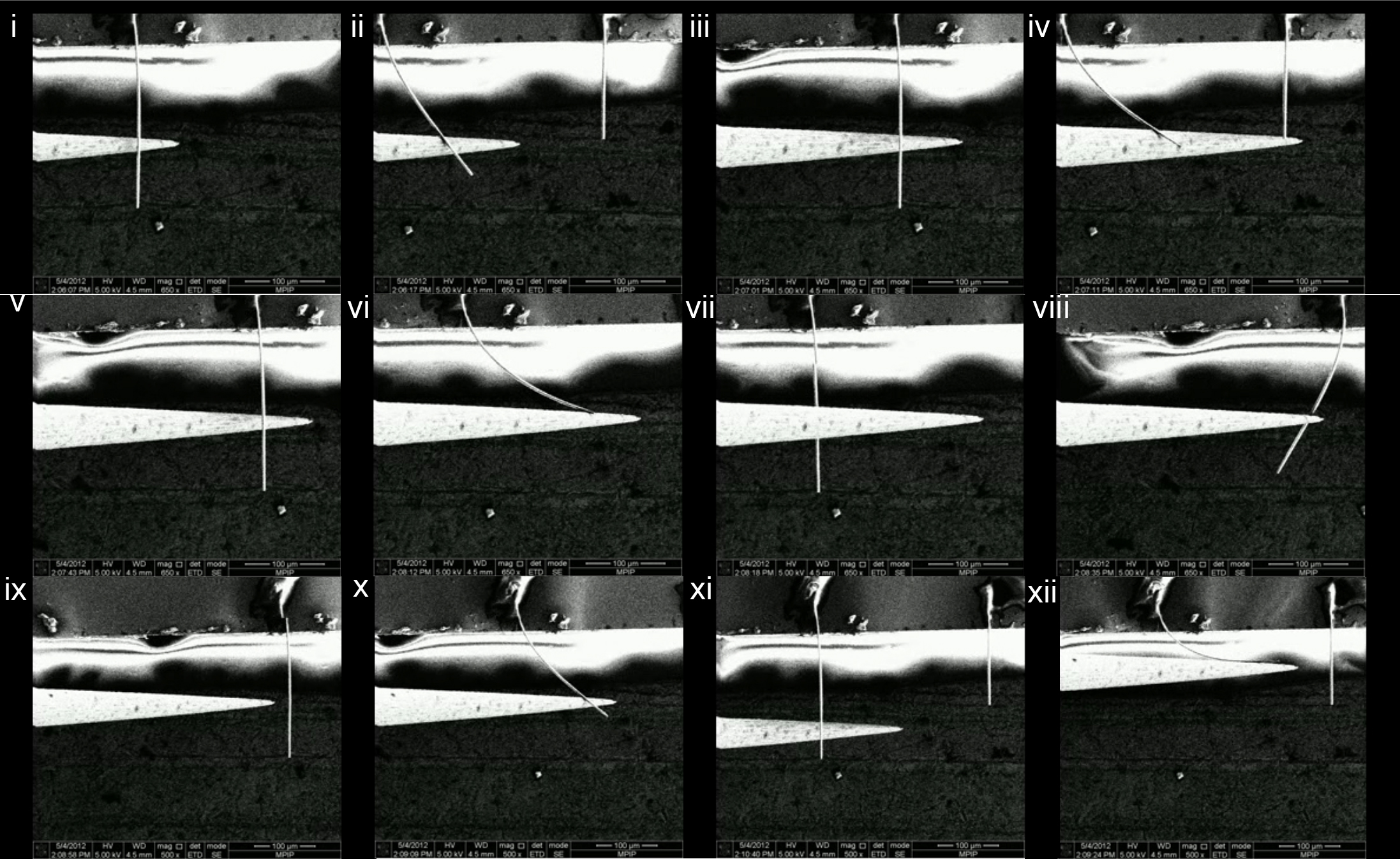


**Figure S12.** Sequence of images showing several bending cycles of immobilized *S. domuncula* spicules (Demospongiae) heat-treated at 500ºC for 1h. It can be observed that after several cycles, the spicule retains its extreme bending properties and returns to original position. There are no signs of plastic deformation or structural fatigue.

**Figure S13.** SEM image of an immobilized *S. domuncula* spicule (Demospongiae) taken out of a series of images recorded during a breakage test, giving the maximum bending before failure. The radius of curvature of the spicule indicated by the white circle was 36 µm, while the spicule diameter was 5.2 µm. This corresponds to a maximum strain of 7.2% before failure.


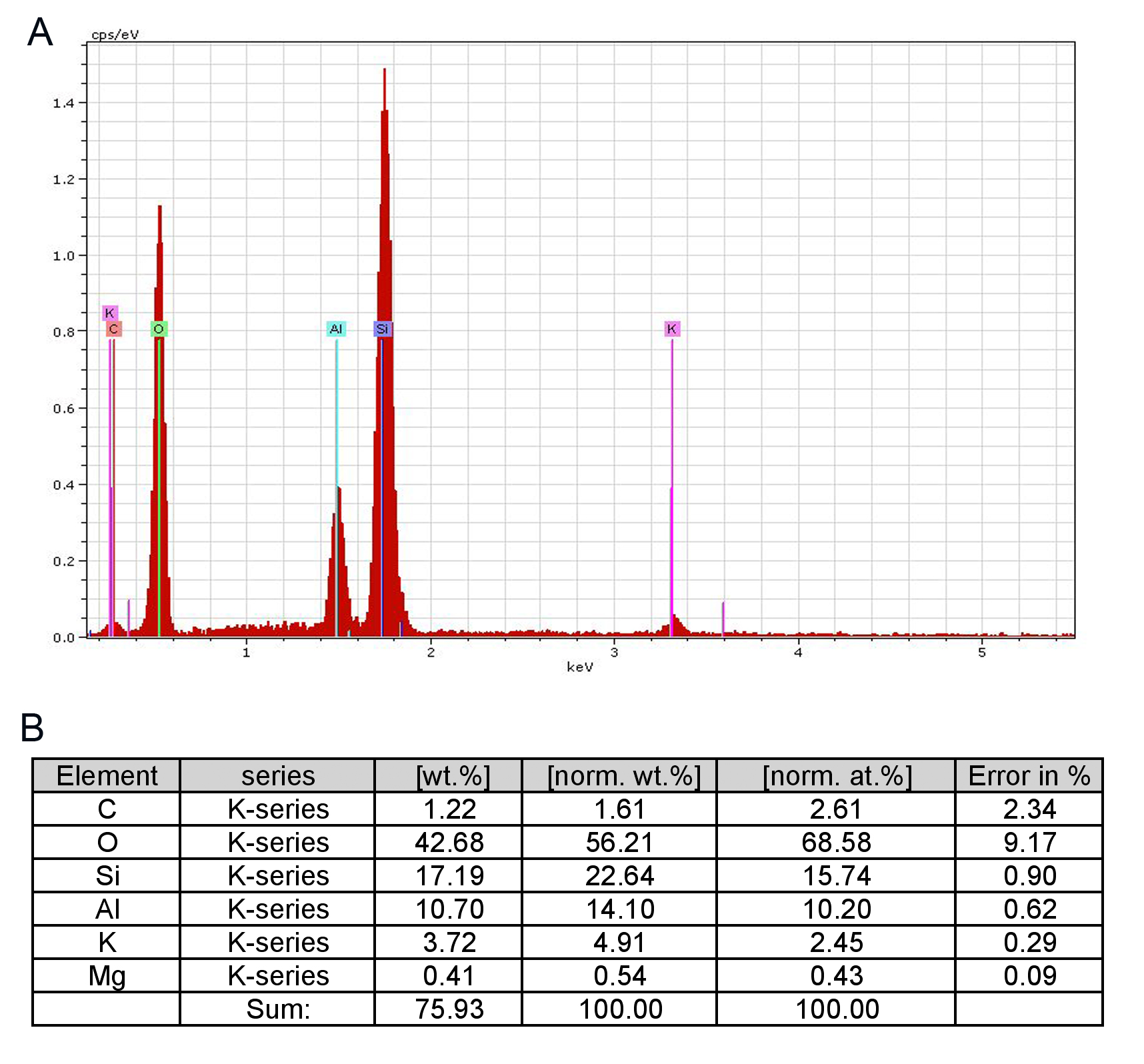


**Figure S14.** Chemical composition of clay used for the experiments. Determination of the chemical elements was performed by EDX. **A** Representative EDX spectra. **B** Summary of chemical composition.

**
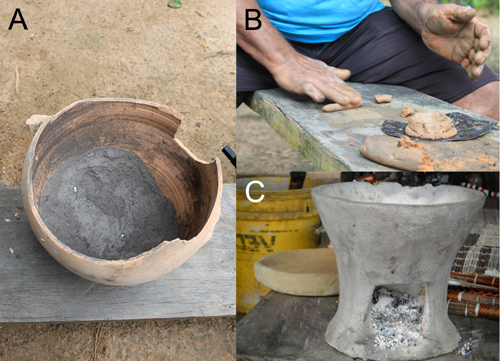
**

**Figure S15. Traditional pre-colonial ceramic techniques.** Digital camera photography taken *in situ* during expedition February 2014. **A** burned cauíxi ready for use. **B** Amazonian natives while performing roll-coil technique with cauíxi. **C** end product of clay and cauíxi still used as spit and known to prevent crack formation/propagation when heated.


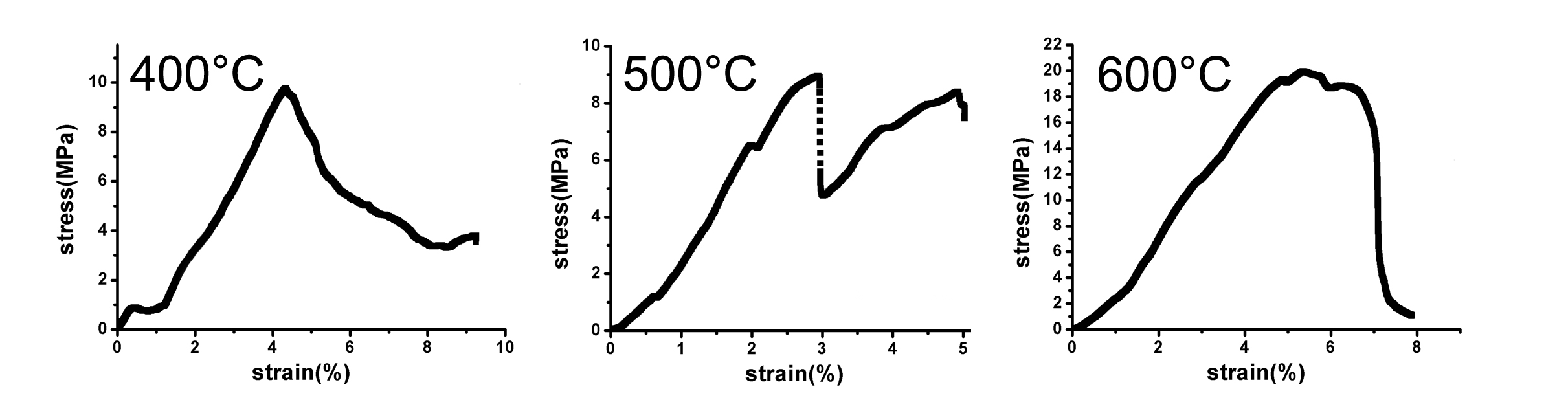


(added)

**Figure S16.** Representative stress-strain curves (1 out of 3 measurements) for ceramic rods after heat treated at different temperature: 400°C, 500°C and 600°C. The assays were done in triplicate. To determine the Young’s modulus the slope values from the elastic section were averaged.

**References**

1. F. Sandford, “Physical and chemical analysis of the siliceous skeletons in six sponges of two groups (demospongiae and hexactinellida)“. *Microsc. Res. Tech.*  **62**, 336-355 (2003).

2. D. W. Schwab, R. E. Shore, “Fine structure and composition of a siliceous sponge spicule”. *Bio. Bull*. **140**, 125-136 (1971).

3. W. E. G. Müller, Sponges (Porifera), Vol. 37, Springer 2003.

4. J. L. Hutter, J. Bechhoefer, “Calibration of atomic-force microscope tips”. *Rev. Sci. Instrum.* **64**, 1868-1873 (1993).

5. F. Natalio *et al.*, “Flexible Minerals: Self-Assembled Calcite Spicules with Extreme Bending Strength”. *Science* **339**, 1298-1302 (2013).
